# The Results of Stomach Surgery

**Published:** 1910-09-10

**Authors:** 


### Surgery.

### THE RESULTS OF STOMACH SURGERY.

There is not the least doubt that cases of gastric and duodenal ulcers, particularly those which have led to actual stenosis either of the pylorus or of the duodenum, are immensely benefited, and sometimes entirely cured, by the operation of gastro-jejunostomy. It is also undoubtedly true that the operation is performed in many cases in which it is not essentially indicated. It makes a considerable difference whether the operator himself or some other person judges of the benefits that accrue whether cases become classed under the heading " cure " or merely under that of " relief "; hence it is very difficult, indeed, to formulate one's own mind upon the question from published statistics; it is only when one sees the patients for oneself that one can really learn the extent to which the so-called cures are complete. Without for one minute denying that absolute cures do result from gastro-jejunostomy for non-malignant conditions of the stomach, and also for the moment neglecting the small, but definite, immediate mortality of the operation, it is probable that more cases are relieved than cured, and that this is so should be borne in mind when a patient is being advised to undergo the surgical treatment. It is not only in Great Britain that impartial observers have come to this conclusion. The results are the same in France, where careful and judicial analyses show clearly that too much has been expected of the operation if the patient is told that he will probably have his symptoms entirely cured.

### Results of Gastro-jejunostomy.

The following instance will perhaps help to illustrate the not uncommon result:—

The patient was a worker in bronze, aged forty-seven, who was operated upon in June 1906 for stenosis of the pylorus caused by the scarring of an old ulcer, the operation performed being gastro-enterostomy. The immediate results were excellent —the gastric symptoms entirely subsided, the appetite was good, and the patient left the hospital well. His report was headed " cured." A few weeks after he had resumed his ordinary mode of living he began to suffer from pain coming on after fatigue or the use of coarse food; this pain generally began about four hours after food, and was situated in the pit of the stomach, radiating thence through to between the shoulders. There was no regurgitation of food or vomiting. He was strongly advised to be more careful in his diet, but two months later the pain was even more severe, and it was then accompanied by vomiting of sour, watery fluid containing a large amount of hydrochloric acid.

Treatment by bismuth and by careful modification of the diet was resorted to, and the man had since kept fairly well though still needing medicinal treatment periodically. He has learnt the need of being prudent, and he knows that he will suffer pain if he takes any liberties with his diet or even if he becomes over-fatigued.

Of all the symptoms from which these patients suffer sooner or later after operation, pain is the most common and the most troublesome. As a rule vomiting is completely cured, though simple regurgitation of sour fluid may occur from time to time independently of pain; this watery fluid contains a little bile as a rule, but seldom much food. Pain, on the other hand, may be severe; it is absent for a few weeks or more, then tends to recur—at first slight and localised, but afterwards more severe and extended over a wider area. Constipation is frequent but slight, and as a rule has been lessened by the operation. If diarrhœa was present before the operation it generally persists and requires regulation of diet and avoïdance of fatigue. Quite apart from the occurrence of any of these symptoms singly, and without there being sufficient evidence upon which to base the diagnosis of the recurrence of the old ulcer, or the development of a new one, there seems to be a special form of dyspepsia that is apt to develop after the operation, characterised by epigastric pain and pain between the shoulders, waterbrash, constipation, and impairment of the general health at least. Many attacks are due no doubt to the unfavourable conditions of life and to the abuse of alcohol or tea that are common amongst the class of patients who come to hospital.

### Recurrent Lesions.

There are abundant records to prove that gastro-jejunostomy does not prevent the formation of fresh ulcers; more or less severe hæmorrhage has occurred after the operation, sometimes when there had been none before it, and in not a few recorded cases such hæmorrhage has proved fatal notwithstanding that gastro-jejunostomy had been previously performed. The operation, moreover, does not remove all risk of cancer, for the latter has been known to occur in the ulcer or even in the scar left after a simple gastric ulcer has been excised. Amongst the 102 cases analysed by Parmentier and Denéchau, 74 were men and 28 women; gastro-enterostomy was the operation performed in all these, except that pylorectomy was done in one case, and in two a perforation was sutured. Three of the patients underwent a second operation, and one was operated upon for carcinoma of the gastro-jejunal opening two years after the original laparotomy. Fifty had been operated on more than four years, the remainder more recently. In some cases the gastro-jejunostomy opening had healed, giving rise to a return of the former symptoms; this was always found to occur only when the anatomical and physiological condition of the pylorus was normal originally, or else had entirely returned to normal. In 7 per cent. the consequences of the operation were bad; in 39 per cent. moderately good—that is to say, there was dyspepsia, but the condition was an improvement upon that which had preceded the operation; and in 54 per cent. it was very fair or good. The most favourable cases were those in which there was organic stenosis; least favourable were those in which the ulcer was far from the pylorus; whilst between the two came those in which the ulcer was near the pylorus but the latter was not stenosed.

### The Importance of Regulating the Diet.

Most of the dyspeptic accidents could be traced to some error of diet, especially the abuse of alcohol or tea. Some patients suffered from the slightest variation of diet and were obliged to keep very strict rules; in those the disease was usually complicated either with disorders due to ptosis of the viscera or with pronounced nervous symptoms.

One of the most striking deductions that can be drawn from the published results of the operation of gastro-jejunostomy for simple gastric ulcer is that one should be exceedingly careful in regulating the diet during convalescence. The operation cannot instantly enable the stomach to carry on its functions normally, and there is too great a tendency to forget this.

